# Predictors and Prognosis of Symptomatic Intracranial Hemorrhage in Acute Ischemic Stroke Patients Without Thrombolysis: Analysis of Data From the Chinese Acute Ischemic Stroke Treatment Outcome Registry

**DOI:** 10.3389/fneur.2021.727304

**Published:** 2021-09-28

**Authors:** Zhiyuan Shen, Haiqiang Jin, Yuxuan Lu, Wei Sun, Ran Liu, Fan Li, Junlong Shu, Liwen Tai, Guozhong Li, Huisheng Chen, Guiru Zhang, Lei Zhang, Xuwen Sun, Jinhua Qiu, Yan Wei, Weiping Sun, Yining Huang

**Affiliations:** ^1^Department of Neurology, Peking University First Hospital, Beijing, China; ^2^Department of Neurology, Second Hospital of Hebei Medical University, Shijiazhuang, China; ^3^Department of Neurology, First Affiliated Hospital of Harbin Medical University, Harbin, China; ^4^Department of Neurology, General Hospital of Shenyang Military Command, Shenyang, China; ^5^Department of Neurology, Penglai People's Hospital, Penglai, China; ^6^Department of Neurology, Fifth Affiliated Hospital of Sun Yat-sen University, Zhuhai, China; ^7^Department of Neurology, Qindao University Medical College Affiliated Yantai Yuhuangding Hospital, Yantai, China; ^8^Department of Neurology, Huizhou First Hospital, Huizhou, China; ^9^Department of Neurology, Harrison International Peace Hospital, Hengshui, China

**Keywords:** ischemic stroke, symptomatic intracranial hemorrhage, risk factors, prognosis, atrial fibrillation, tumor

## Abstract

**Background and Purpose:** There is limited information on symptomatic intracranial hemorrhage (sICH) in stroke patients without thrombolysis. This study aimed to evaluate the risk factors of sICH and the association between sICH and the prognosis at 3 and 12 months in acute ischemic stroke patients without thrombolysis.

**Methods:** Data originated from the Chinese Acute Ischemic Stroke Treatment Outcome Registry. Univariate analysis and multivariate logistic regression were used to screen the risk factors of sICH. Multivariable logistic regression models were used to assess the association of sICH with poor outcome and all-cause mortality.

**Results:** Totally, 9,484 patients were included, of which 69 (0.73%) had sICH. Atrial fibrillation (odds ratio [OR], 3.682; 95% confidence interval [CI], 1.945–6.971; *p* < 0.001), history of tumors (OR, 2.956; 95% CI, 1.115–7.593; *p* = 0.024), and the National Institutes of Health Stroke Scale (NIHSS) score on admission ([6–15: OR, 2.344; 95% CI, 1.365–4.024; *p* = 0.002] [>15: OR, 4.731; 95% CI, 1.648–13.583; *p* = 0.004]) were independently associated with sICH. After adjustment of the confounders, patients with sICH had a higher risk of poor outcome (OR, 1.983; 95% CI, 1.117–3.521; *p* = 0.018) at 3 months and that of all-cause mortality at 3 (OR, 6.135; 95% CI, 2.328–16.169; *p* < 0.001) and 12 months (OR, 3.720; 95% CI, 1.513–9.148; *p* = 0.004).

**Conclusion:** sICH occurred in 0.73% of acute ischemic stroke patients without thrombolysis and was associated with a worse prognosis at 3 and 12 months. Atrial fibrillation, history of tumors, and NIHSS score at admission were independent risk factors of sICH.

## Introduction

Stroke is the second leading cause of disability-adjusted life years worldwide in people over 50 years of age (1), which has brought a heavy burden on society and families. Until now, intravenous thrombolysis with recombinant tissue plasminogen activator (rt-PA) remains the first line treatment for patients with acute ischemic stroke. Symptomatic intracranial hemorrhage (sICH) is recognized as a devastating complication of thrombolysis treatment, which occurs in 0.4–10.3% patients depending on the varied diagnostic criteria and is consistently associated with an increased mortality and a worse functional outcome (2–16). Many studies have reported the predictors of sICH after intravenous thrombolysis, including stroke severity, age, onset-to-treatment time, baseline glucose, hyperdense cerebral artery sign, and early infarct signs on baseline imaging (2, 3, 7, 17). However, there is limited information on sICH in stroke patients without intravenous thrombolysis.

Although the incidence of sICH was lower in the patients who received placebo compared to those given t-PA in the National Institute of Neurological Disorders and Stroke (NINDS) trial (0.6 vs. 6.4%), management of acute stroke remains challenging, considering the vast number of stroke patients without intravenous thrombolysis worldwide (18). Tan et al. (19) reported that 4.4% of ischemic stroke patients without thrombolysis developed sICH. But this is a single-center study with only 406 patients included. Another study based on a multicenter registry analyzed sICH in those who did not receive any antithrombotic therapy (20), while most patients with acute ischemic stroke would receive antithrombotic treatment after stroke, of which approximately one third underwent antiplatelet therapy (APT) prior to the onset of stroke in the real world (21, 22).

The purpose of this study is to investigate the risk factors and prognosis of sICH in patients with acute ischemic stroke that did not undergo thrombolytic therapy in a large multicenter, prospective cohort in China.

## Methods

### Study Design and Population

Data was obtained from the Chinese Acute Ischemic Stroke Treatment Outcome Registry (CASTOR), a multicenter, prospective, hospital-registry (*n* = 80) study conducted in 46 cities across China. The trial design and protocol were described elsewhere (23). The hospitals included in our study were required to have a neurology ward with over 100 stroke patients admitted every year. Consecutive patients from May 2015 to October 2017 were eligible for enrollment in the study if they met the following criteria: (1) age ≥18 years. (2) acute ischemic stroke diagnosed according to the Chinese Guideline for Diagnosis and Treatment of Ischemic Stroke (2014). (3) admitted within 1 week after onset of stroke. (4) consent to participation in this study. Patients with cerebral hemorrhage or an expected survival <3 months due to systemic diseases were excluded. Patients were assessed five times during the course of the study at admission, 7 ± 2 days after enrollment, discharge, and ~3- and 12- months post-stroke.

This study was registered with ClinicalTrials.gov (NCT02470624) and approved by the ethics committees of Peking University First Hospital (IRB approval number: 2015[922]) and all participating hospitals. This study was conducted in accordance with the International Conference on Harmonization Good Clinical Practice (ICH-GCP) guidelines and the Declaration of Helsinki. Written informed consent was obtained from all patients or an guardian (if the patient was unable to provide it) to participate.

### Data Collection

Baseline information was collected predominantly by face-to-face interviews. The details of the diagnosis and treatment strategy during admission were obtained from the medical records and interviews with patients or their proxies. The information included: (1) demographic variables, including age and sex; (2) medical history, including hypertension (patients taking antihypertensive agents or with blood pressure >140/90 mmHg on repeated measurements), diabetes mellitus (patients taking antidiabetic agents, with fasting blood sugar level >126 mg/dL or HbA1c ≥6.5%, or with a casual plasma glucose level >200 mg/dL), hyperlipidemia (patients taking lipid-lowering agents or with an overnight fasting cholesterol level >240 mg/dL, triglyceride level >200 mg/dL, or low-density lipoprotein level >160 mg/dL), history of stroke (previous cerebral infarction and/or hemorrhage), atrial fibrillation(AF), coronary heart disease, history of tumors; (3) medication history within 3 months prior to onset of stroke (single antiplatelet agents, dual antiplatelet agents, lipid-lowering agents, antihypertensive agents, antidiabetic agents); (4) medication administered after onset of stroke (thrombolysis, antiplatelet agents, anticoagulation agents, lipid-lowering agents, antihypertensive agents, antidiabetic agents); (5) clinical features of the index stroke, including National Institutes of Health Stroke Scale score (NIHSS), Glasgow Coma Scale score (GCS) and systolic and diastolic blood pressure on admission.

### Diagnosis of sICH

Repeated brain CT/MRI was suggested when neurological deterioration occurred in stroke patients. sICH was defined as the hemorrhage confirmed by CT/MRI scans during admission with clinical deterioration or an increase of four or more points in NIHSS score or adverse events indicating clinical worsening (e.g., drowsiness, increase of hemiparesis) recorded by the investigator, according to the definitions outlined in the European-Australasian Acute Stroke Study (ECASS-II) classification (24).

### Outcome Assessment

The functional outcome measured using the modified Rankin scale (mRS) was collected via face-to-face or telephone interview at 3 and 12 months after the onset of symptoms. Poor outcome was defined as a mRS score of 3–6. All-cause death was defined as death from any cause and confirmed by a death certificate from the hospital or the local citizen registry. The outcomes in our study included the proportion of poor outcome and all-cause mortality at 3 and 12 months.

### Statistical Analysis

Data were expressed as median values, inter-quartile ranges (IQR) for continuous variables, and frequencies and percentages for discrete variables. The statistical significance of intergroup differences was assessed using Mann-Whitney *U*-test or χ^2^ tests as appropriate. Multivariate logistic regression analysis was subsequently used to identify the independent risk factors from those variables with *p* < 0.1 in the univariate analysis. Calculated odds ratios (ORs) were used to measure the association between sICH and risk factors. The relationship of sICH with poor outcome and all-cause mortality was assessed using several logistic regression models. Model 1 was adjusted for age and sex; Model 2 was further adjusted for medical history (previous stroke, hypertension, diabetes mellitus, dyslipidemia, coronary heart disease, AF, and history of tumors), medication 3 months prior to the onset of stroke (lipid-lowering agents antiplatelet agents, antihypertensive agents), and the clinical features of the index stroke (NIHSS and GCS scores on admission, diastolic pressure, and systolic pressure on admission) based on Model 1 and Model 3 was further adjusted for treatment in the hospital (antidiabetic agents, antihypertensive agents, lipid-lowering agents, antithrombotic agents) based on Model 2. A sensitivity analysis was performed that restricted the study population to those who were admitted within 48 h of stroke onset. All *p*-values were two-sided, with *p* < 0.05 considered statistically significant. All statistical analyses were performed using SPSS version 25.0 (SPSS Inc., Chicago, IL, USA).

## Results

### Patient Characteristics and Incidence of sICH

**Figure 1:**
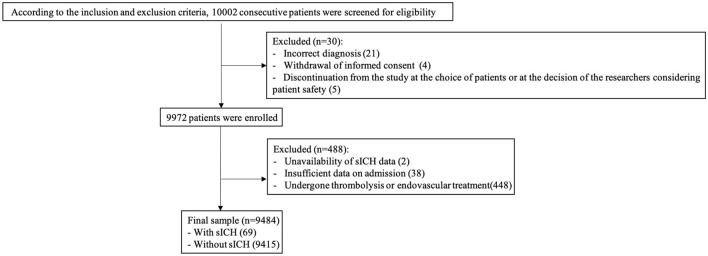
Study patients flow chart.

In total, 10,002 consecutive patients with ischemic stroke within 7 days of onset were enrolled in the CASTOR study. We excluded 518 patients for the following reasons: incorrect diagnosis (*n* = 21), withdrawal of informed consent (*n* = 4), discontinuation from the study at the choice of patients or at the decision of the researchers considering patient safety (*n* = 5), unavailability of sICH data (*n* = 2), insufficient data on admission (*n* = 38), and undergoing intravenous thrombolysis or endovascular treatment (*n* = 448). Finally, 9,484 patients (median age, 64.0 years; 65.6% males) were included in this the analysis (Figure 1). The median NIHSS score at admission was 4 (IQR 2–7). Among the 9,484 patients, 69 cases (0.73%; median age, 66.0 years; 66.7% males) had developed sICH during admission.

### Predictors of sICH

The characteristics of patients with and without sICH are summarized in Table 1. Univariate analysis revealed the differences between patients with and without sICH were significant in the following features: age (*p* = 0.012), NIHSS score at admission (*p* < 0.001), GCS score at admission (*p* < 0.001), AF (*p* < 0.001), history of tumors (*p* = 0.012), antidiabetic agents during admission (*p* = 0.026), and antithrombotic agents during admission (*p* = 0.006). The multivariate logistic regression analysis showed that AF (OR, 3.682; 95% CI, 1.945–6.971; *p* < 0.001), history of tumors (OR, 2.956; 95% CI, 1.115–7.593; *p* = 0.024), and NIHSS score on admission ([6–15: OR, 2.344; 95% CI, 1.365–4.024; *p* = 0.002] [>15: OR, 4.731; 95% CI, 1.648–13.583; *p* = 0.004]) were the independent risk factors of sICH (Table 2).

**Table 1:** Baseline characteristics of ischemic stroke patients with and without sICH (*n* = 9,484).

	**All**	**Without sICH (*n* = 9,415)**	**With sICH (*n* = 69)**	***P-*values**
Male	6,221(65.6)	6,175(65.6)	46(66.7)	0.851
Age	64.0(55.0–72.0)	64.0(55.0–72.0)	66.0(59.5–75.0)	**0.012**
Diastolic pressure on admission	87(80–97)	87(80–97)	89(76–98)	0.943
Systolic pressure on admission	150(135–164)	150(135–164)	155(136–167)	0.259
NIHSS score on admission				** <0.001**
0–5	6,067(64.0)	6,041(64.2)	26(37.7)	
6–15	2,929(30.9)	2,899(30.8)	30(43.5)	
>15	488(5.1)	475(5.0)	13(18.8)	
GCS score on admission				** <0.001**
13–15	8,594(90.6)	8,540(90.7)	54(78.3)	
9–12	691(7.3)	683(7.3)	8(11.6)	
3–8	199(2.1)	192(2.0)	7(10.1)	
**Past history**				
Previous stroke	2,258(23.8)	2,243(23.8)	15(21.7)	0.685
Hypertension	6,141(64.8)	6,101(64.8)	40(58.0)	0.237
Diabetes mellitus	2,460(25.9)	2,436(25.9)	24(34.8)	0.093
Dyslipidemia	296(3.1)	294(3.1)	2(2.9)	0.915
Coronary heart disease	1,315(13.9)	1,303(13.8)	12(17.4)	0.395
Atrial fibrillation	515(5.4)	499(5.3)	16(23.2)	**<** **0.001**
History of tumors	238(2.5)	233(2.5)	5(7.2)	**0.012**
**Medication before admission (3 months prior to stroke)**				
Lipid-lowering agents	422(4.4)	417(4.4)	5(7.2)	0.258
Antiplatelet agents	0.575			
None	8,641(91.1)	8,576(91.1)	65(94.2)	
Single antiplatelet agents	728(7.7)	725(7.7)	3(4.3)	
Dual antiplatelet agents	115(1.2)	114(1.2)	1(1.4)	
Antihypertensive agents	2,853(30.1)	2,837(30.1)	16(23.2)	0.210
**Treatment in hospital**				
Antidiabetic agents	2,825(29.8)	2,796(29.7)	29(42.0)	**0.026**
Antihypertensive agents	4,308(45.4)	4,275(45.4)	33(47.8)	0.688
Lipid-lowering agents	8,684(91.6)	8,620(91.6)	64(92.8)	0.721
Antithrombotic agents				**0.006**
None	409(4.3)	402(4.3)	7(10.1)	
Antiplatelet agents	7,783(82.1)	7,737(82.2)	46(66.7)	
Anticoagulant agents	133(1.4)	131(1.4)	2(2.9)	
Antiplatelet + anticoagulant agents	1,159(12.2)	1,145(12.2)	14(20.3)	

*Values are reported as n (%) or as Median (interquartile range)*.

*sICH, symptomatic intracranial hemorrhage; NIHSS, National Institutes of Health Stroke Scale; GCS, Glasgow Coma Scale*.

*p-values in bold fonts indicate significant associations*.

**Table 2:** Multivariate analysis to identify factors associated with sICH in patients with ischemic stroke without thrombolysis.

	**OR**	**95%CI**	***P-*values**
Atrial fibrillation	3.682	1.945–6.971	** <0.001**
History of tumors	2.956	1.115–7.593	**0.024**
NIHSS score on admission			
0–5	*Ref*.		
6–15	2.344	1.365–4.024	**0.002**
>15	4.731	1.648–13.583	**0.004**

*NIHSS, National Institutes of Health Stroke Scale; OR, odds ratio; CI, confidence interval*.

*p-values in bold fonts indicate significant associations*.

### Antithrombotic Therapy and sICH in Patients With AF

In our study, 515 patients (5.4%) had a history of AF, of which 250 (48.5%) patients received antiplatelet therapy, 71 (13.8%) received anticoagulant therapy, 162 (31.5%) received both antiplatelet and anticoagulant therapy, and 32 (6.2%) did not receive any antithrombotic therapy during admission. In patients with AF, the development of sICH was not significantly associated with the antithrombotic regimen (Table 3).

**Table 3:** Antithrombotic regimens and sICH in patients with atrial fibrillation.

	**All**	**Without sICH (*n* = 499)**	**With sICH (*n* = 16)**	***P-*values**
Antithrombotic agents				0.141
None	32(6.2)	29(5.8)	3(18.8)	
Antiplatelet agents	250(48.5)	244(48.9)	6(37.5)	
Anticoagulant agents	71(13.8)	70(14.0)	1(6.3)	
Antiplatelet + anticoagulant agents	162(31.5)	156(31.3)	6(37.5)	

*sICH, symptomatic intracranial hemorrhage*.

### Tumor Types and sICH

In our analysis, 238 (2.5%) patients had a history of tumors, of which 10 (42%) had nasopharyngeal cancer, 35 (14.7%) had malignant tumors of the digestive system, 14 (5.9%) had lung cancer, 15 (6.3%) had breast cancer, 8 (3.4%) had liver cancer, 33 (13.9%) had reproductive system tumors, 5 (2.1%) had tumors of the hematological system, 8 (3.4%) had a combination of two types of tumors, and 110 (46.2%) had other tumors. There was no significant association between tumor types and sICH (*p* = 0.251) (Table 4).

**Table 4:** Tumor type and sICH in acute ischemic stroke patients without thrombolysis.

	**All**	**Without sICH (*n* = 233)**	**With sICH (*n* = 5)**	***P-*values**
Nasopharyngeal cancer	10(4.2)	10(4.3)	0(0.0)	0.251
Malignant tumors of the digestive system	35(14.7)	33(14.2)	2(40.0)	
Lung cancer	14(5.9)	15(6.0)	0(0.0)	
Breast cancer	15(6.3)	15(6.0)	1(20.0)	
Liver cancer	8(3.4)	7(3.0)	1(20.0)	
Reproductive system tumors	33(13.9)	33(14.2)	0(0.0)	
Hematological system tumors	5(2.1)	5(2.1)	0(0.0)	
Other tumors	110(46.2)	119(46.8)	1(20.0)	
Combined with two types of tumors	8(3.4)	8(3.4)	0(0.0)	

*sICH, symptomatic intracranial hemorrhage*.

### Prior APT and sICH

Among the 9,484 patients without thrombolysis, 843 (8.9%) received APT within 3 months prior to stroke onset, of which 115 received dual APT treatment with aspirin and clopidogrel, and 728 received single APT (663 patients with aspirin alone, 117 with clopidogrel alone, and 5 with cilostazol alone). There was no significant difference in the risk of sICH among the three groups (0.8% in the no APT group, 0.4% in the single APT group and 0.9% in the dual APT group, *p* = 0.575).

### sICH and Poor Outcome

The mRS score at 3 months was collected in 8,890 (93.7%) patients, of which 1,734 (19.5%) had a mRS score of 3–5 and 95 (1.1%) patients had died (mRS = 6). At 12 months post stroke onset, the mRS score was collected in 8,332 (87.9%) patients, of which 1,209 (12.7%) had an mRS score 3–5 and 191(2.0%) patients had died. Compared to those without sICH, the patients with sICH had a higher risk of poor outcome (45.3 vs. 20.4%, *p* < 0.001; 33.3 vs. 16.7%, *p* = 0.001, respectively) and mortality (10.9 vs. 1.0%, *p* < 0.001; 13.3 vs. 2.2%, *p* < 0.001, respectively) at 3 and 12 months. After adjusting for the confounding variables, the differences in poor outcome (OR, 1.983; 95% CI, 1.117–3.521; *p* = 0.018) at 3 months and all-cause mortality at 3 (OR, 6.135; 95% CI, 2.328–16.169; *p* < 0.001) and 12 months (OR, 3.720; 95% CI, 1.513–9.148; *p* = 0.004) remained statistically significant (Table 5).

**Table 5:** Relationship of all-cause death and poor functional prognosis with sICH in patients without thrombolysis.

**Outcomes**	**OR**	**95%CI**	***P-*values**
At 3 months			
Poor outcome			
Unadjusted	3.234	1.972–5.305	<0.001
Model 1	3.019	1.824–4.997	<0.001
Model 2	2.000	1.128–3.549	0.018
Model 3	1.983	1.117–3.521	0.019
Death			
Unadjusted	12.194	5.411–27.482	<0.001
Model 1	10.069	4.337–23.377	<0.001
Model 2	6.711	2.651–16.992	<0.001
Model 3	6.135	2.328–16.169	<0.001
At 12 months			
Poor outcome			
Unadjusted	2.497	1.455–4.284	0.001
Model 1	2.284	1.307–3.989	0.004
Model 2	1.530	0.832–2.812	0.171
Model 3	1.550	0.842–2.855	0.159
Death			
Unadjusted	6.800	3.185–14.521	<0.001
Model 1	5.725	2.576–12.726	<0.001
Model 2	3.718	1.540–8.979	0.004
Model 3	3.720	1.513–9.148	0.004

*Model 1: adjusted for age and sex*.

*Model 2: adjusted for age, sex, medical history (previous stroke, hypertension, diabetes mellitus, dyslipidemia, coronary heart disease, AF, and history of tumors), medication 3 months prior to the onset of stroke (lipid-lowering agents, antiplatelet agents, antihypertensive agents), and the clinical features of the index stroke (NIHSS and GCS scores on admission, diastolic pressure and systolic pressure on admission)*.

*Model 3: adjusted for variables in model 2, plus treatment in the hospital (antidiabetic agents, antihypertensive agents, lipid-lowering agents, antithrombotic agents)*.

*OR, odds ratio; CI, confidence interval; mRS, modified Rankin scale*.

### Sensitivity Analysis

When we restricted the study population to those who were admitted within 48 h of stroke onset, there were 6,835 patients were included in the sensitivity analysis, of which 55 (0.8%) patients developed sICH. AF (OR, 3.432; 95% CI, 1.723–6.835; *p* < 0.001), history of tumors (OR, 3.255; 95% CI, 1.128–9.391; *p* = 0.029), and NIHSS score at admission ([6–15: OR, 2.844; 95% CI, 1.524–5.307; *p* = 0.001] [>15: OR, 5.073; 95% CI, 1.601–16.072; *p* = 0.006]) were the independent predictors of sICH (Supplementary Tables 1–4). Further, sICH was significantly associated with the increased risk of poor outcome at 3 months and all-cause mortality at 3 and 12 months (Table 6).

**Table 6:** Sensitivity analysis.

**Outcomes**	**OR**	**95%CI**	***P-*values**
At 3 months			
Poor outcome			
Unadjusted	3.632	2.091–6.310	<0.001
Model 1	3.449	1.966–6.053	<0.001
Model 2	2.185	1.159–4.121	0.016
Model 3	2.148	1.138–4.053	0.018
Death			
Unadjusted	11.678	4.830–28.237	<0.001
Model 1	9.923	3.967–24.823	<0.001
Model 2	5.671	2.038–15.783	0.001
Model 3	5.285	1.813–15.403	0.002
At 12 months			
Poor outcome			
Unadjusted	2.818	1.565–5.073	0.001
Model 1	2.626	1.430–4.822	0.002
Model 2	1.661	0.857–3.218	0.133
Model 3	1.688	0.869–3.279	0.122
Death			
Unadjusted	6.990	3.083–15.849	<0.001
Model 1	5.927	2.484–14.140	<0.001
Model 2	3.786	1.455–9.851	0.006
Model 3	3.756	1.417–9.961	0.008

*Patients admitted within 48 h after the onset of stroke were included in the sensitivity analysis (n = 6,835)*.

*Model 1: adjusted for age and sex*.

*Model 2: adjusted for age, sex, medical history (previous stroke, hypertension, diabetes mellitus, dyslipidemia, coronary heart disease, AF, and history of tumors), medication 3 months prior to the onset of stroke (lipid-lowering agents, antiplatelet agents, antihypertensive agents), and the clinical features of the index stroke (NIHSS and GCS scores on admission, diastolic pressure and systolic pressure on admission)*.

*Model 3: adjusted for variables in model 2, plus treatment in the hospital (antidiabetic agents, antihypertensive agents, lipid-lowering agents, antithrombotic agents)*.

*OR, odds ratio; CI, confidence interval; mRS, modified Rankin scale*.

## Discussion

In this study, we found that sICH occurred in 0.73% of acute ischemic stroke patients without thrombolysis during admission and AF, history of tumors, and NIHSS score at admission were the independent risk factors of sICH. In these patients, sICH was associated with a higher risk of poor outcome at 3 months and an increased mortality at 3 and 12 months. No significant association between sICH and poor outcome at 12 months was observed.

Previous studies on acute ischemic stroke have focused on hemorrhagic transformation (HT), while the diagnosis of sICH required an imaging change and a deterioration in neurological function. In our study, the incidence of sICH in patients without thrombolysis was similar to that noted in NINDS trial (0.73 vs. 0.6%) (18). Several studies have reported a higher incidence of sICH in patients without thrombolysis. A systematic review showed that the incidence of sICH was 1.5% (4), which may be attributed to differences in patient selection criteria, the diagnostic criteria of sICH, the time interval between stroke onset and admission, and stroke treatment. Two studies from West China Hospital reported that the incidence of sICH was 1.3% in 2010–2011 and 4.4% in 2002–2005, respectively (15, 19). There were more patients with mild stroke in our cohort, which may explain this discrepancy. In addition, the sample sizes of these two studies were relatively small, and the patients were recruited from a single center.

We found AF was associated with an ~4-fold increase in the risk of sICH in the patients without thrombolysis. A similar result was reported by Tan et al. (19) In patients with thrombolysis, AF was also recognized as an independent risk factor of sICH with the OR ranging from 2.5 to 7 (25). Patients with cardiogenic stroke usually have rapid occlusion of arteries, less developed cerebral collateral circulation, small penumbra and large core infarction, which increases HT (26, 27). Most patients with AF may receive anticoagulant therapy, but previous trials have shown that anticoagulation could increase the risk of intracerebral bleeding in ischemic stroke patients (28). However, Lee et al. (29) reported that the incidence of sICH did not increase in patients with cardiogenic embolism who received early anticoagulation therapy within 1 week from stroke onset. Similarly, our analysis did not find a significant association between the antithrombotic regimen during admission and sICH in patients with AF, either. The risk in those with anticoagulation was not higher than that with antiplatelet medication. This finding was also consistent with those of several other studies (30–32), and this indicated that it was AF not the accompanying anticoagulation therapy which caused the increased the risk of sICH.

In this study, a higher NIHSS score at admission was associated with sICH in patients with acute ischemic stroke who did not undergo thrombolysis. Similar results were noted in those with ischemic stroke after thrombolysis (3, 25, 33, 34). In previous studies on patients without thrombolysis, although univariate analysis showed that patients with higher NIHSS score were more prone to HT, NIHSS score was not an independent risk factor for HT (2, 19, 20). This is perhaps explained by the smaller sample sizes of previous studies and the fact that previous studies only investigated the relationship between HT and NIHSS score at admission rather than the relationship between sICH and NIHSS score at admission. Severe ischemic stroke usually manifests as extensive brain tissue damage, including vascular damage, which is prone to bleeding.

Previous studies found that antithrombotic medications before acute ischemic stroke might increase the risk of sICH after intravenous thrombolysis (35, 36). In our study, although patients with prior APT were older and more likely to have vascular risk factors which may increase the risk of sICH, pre-stroke APT did not increased the risk of sICH, which suggests that prior use of APT did not increase the risk of sICH in stroke patients without thrombolysis. Similar results were reported in some other studies (2, 20). A study that included 12,415 patients without thrombolysis found no correlation between APT and HT, although reported a higher proportion of pre-stroke APT (17.54%) (20). The variations in the doses of pre-stroke antiplatelet therapy and the issue of patient compliance were not addressed in our study. Therefore, further research is needed.

Interestingly, we found an association between the history of tumors and sICH in acute ischemic stroke patients without thrombolysis, which had not been reported previously. Several studies investigated the relationship between a history of tumors and sICH after thrombolysis and their results varied greatly. The overall risk of hemorrhagic stroke occurrence in patients with cancer is significantly higher than that in the general population (37). Whether brain hemorrhage is directly or indirectly caused by cancer is not clear. In our study, the initial brain imaging before enrolment could exclude those patients with typical lesions of primary brain tumors or metastatic tumors. However, we could not rule out the possibility that some patients with atypical lesions of tumor had been included in the study and then had hemorrhagic transformation. Other studies indicated severe thrombocytopenia and coagulopathy may be a mechanism of cerebral hemorrhage related to tumor (37). Thus, further research is needed to investigate the mechanisms of sICH in acute ischemic stroke patients with history of tumors. Although in the latest acute ischemic stroke management guidelines, the American Heart Association and the American Stroke Association provided with some suggestions for patients with a history of tumors, such as intravenous thrombolysis is contraindicated in cases of intra-axial intracranial neoplasms and gastrointestinal malignancy, and recommended in cases of extra-axial intracranial neoplasms and systemic malignancy with reasonable (>6 months) life expectancy, the guidelines admitted that the efficacy and safety of the treatments in stroke patients with malignant disease are still unclear (38). With aging and the improvement in tumor therapy, there will be more tumor survivors who are also vulnerable to stroke. Our study showed a higher risk of sICH in stroke patients with a history of tumors, which indicated that there would be some different characteristics in these patients and warranted further research on the management of stroke in patients with history of tumors.

There was a relatively lower mortality (1.1% at 3 months and 2.0% at 12 months) in our study, which was similar to the results based on the Third China National Stroke Registry (1.3% at 3 months and 2.9% at 12 months) (39). Andrade et al. (40) reported a mortality of 6.9% at discharge and Paciaroni et al. (2) reported a mortality of 11.5% at 3 months in stroke patients. This difference may be due to the lower average age of patients and the lower median NIHSS score at admission in our cohort.

A prospective cohort study showed that post-thrombolytic sICH contributed to an increased risk of poor outcome and mortality. According to the different definitions of sICH used, the ranges of OR were 1.3–1.7 and 1.5–4.8, respectively (3). Another multicenter prospective cohort study showed that sICH could increase the risk of poor outcome by 3.57 times (7). Our findings suggested that patients with sICH had a higher risk of poor outcome at 3 months. Additionally, the patients with sICH still had a higher mortality rate at 3 and 12 months and it revealed sICH in stroke patients without thrombolysis was a serious problem in management of stroke since most patients did not receive thrombolysis in the real world. We speculated two possible reasons that would contribute to the worse prognosis. One was the direct influence of sICH, and the other reason was that the withdrawal of antithrombotic therapy at the early stage of treatment due to sICH increased the recurrent ischemic stroke. In our study, patients with sICH had a higher incidence of poor outcome at 12 months than those without sICH (33.3 vs. 16.7%, *p* = 0.001). However, the difference became insignificant after adjustment of the baseline characteristics and treatment during admission. Considering the mRS score was not collected in 1,152 patients at 12 months, we could not rule out the possibility of selective dropout.

The strengths of our study include a relatively large sample size and a multicenter design. However, our study has several limitations that need to be addressed. First, this study was an observational investigation, we adjusted for a series of identified confounding variables. However, due to the nature of observational studies, certain unmeasured or residual confounding effects were unavoidable. Therefore, we cannot conclude a causal relationship between sICH and poor outcome. Second, repeated brain imaging was often performed when the symptoms of stroke patients worsen in clinical practice, which may underestimate the risk of ICH, especially in the patients with asymptomatic or mild symptoms. However, our study focused on the patients with sICH, who had a deterioration in neurological function accompanying with ICH. So we think the risk of underestimation was limited in our study. Third, some information such as glucose level at admission, blood pressure during admission, details of brain imaging including radiographic classification of HT, and number of cerebral microbleeds were not collected in the database. So our study propably missed to elucidate the responsible mechanisms and potential protective measures of sICH. Fourth, the exact time of sICH was not recorded in our database. While sICH occurring at different timepoints may be due to varied pathological mechanisms. Fifth, although we found an association between history of tumors and sICH, the limited sample with tumor needed the further confirmation. Sixth, we did not include the patients who underwent endovascular treatment although thrombectomy treatment had been recommended in the current guidelines. Considering there were only 23 patients who received endovascular treatment in our study and the significant difference in the risk of bleeding, we focused on those who only received medication treatment. Seventh, the CASTOR study recruited the patients who were admitted within 7 days of onset from acute ischemic stroke, which indicates a possible heterogenous population, and those who had developed sICH at early stage would not be included in this registry, which may underestimate the incidence of sICH. However, in the sensitivity analysis which only included those admitted within 48 h, the results were similar to that of our main analysis. Finally, all the patients were recruited in China. The rate of receiving intravenous thrombolysis and endovascular treatment, as well as the mortality were relatively lower than other studies in the western countries (2, 41–44). Hence, caution is needed when generalizing our results to other populations.

## Conclusions

In a large, multicenter cohort of acute ischemic stroke patients, sICH occurred in 0.73% of patients without thrombolysis and was associated with a worse prognosis at 3 and 12 months post stroke. AF, a history of tumors, and NIHSS score at admission were the independent risk factors of sICH.

## Data Availability Statement

The raw data supporting the conclusions of this article will be made available by the authors, without undue reservation.

## Ethics Statement

The studies involving human participants were reviewed and approved by the ethics committee of Peking University First Hospital (IRB approval number: 2015[922]) and all participating hospitals. The patients/participants provided their written informed consent to participate in this study.

## Author Contributions

WeipS and YH: conceptualization, methodology, supervision, and writing—review and editing. ZS and YL: data curation. HJ, WeiS, RL, FL, JS, LT, GL, HC, GZ, LZ, XS, JQ, and YW: investigation. ZS: writing—original draft. All authors have read and agreed to the published version of the manuscript.

## Funding

This work was supported by the National Natural Science Foundation of China (Grant No. 81400944) and Peking University Medicine Seed Fund for Interdisciplinary Research (BMU2018MX020). The authors declare that this study received funding from Techpool Bio-Pharma Co., Ltd. The funder was not involved in the study design, collection, analysis, interpretation of data, the writing of this article or the decision to submit it for publication.

## Conflict of Interest

The authors declare that the research was conducted in the absence of any commercial or financial relationships that could be construed as a potential conflict of interest.

## Publisher's Note

All claims expressed in this article are solely those of the authors and do not necessarily represent those of their affiliated organizations, or those of the publisher, the editors and the reviewers. Any product that may be evaluated in this article, or claim that may be made by its manufacturer, is not guaranteed or endorsed by the publisher.
