# Supplementary material for: Predictors and Prognosis of Symptomatic Intracranial Hemorrhage in Acute Ischemic Stroke Patients Without Thrombolysis: Analysis of Data From the Chinese Acute Ischemic Stroke Treatment Outcome Registry
Source: Front Neurol. 2021 Sep 28;12:727304. doi: 10.3389/fneur.2021.727304 (PMC8506002; doi:10.3389/fneur.2021.727304)
Supplement: Supplementary file 1 [file Table_1.docx]

Supplementary Material

**Table S1** Baseline characteristics of ischemic stroke patients with and without sICH (n = 6835)

|  | All | Without  sICH (n = 6780) | With  sICH (n = 55) | *P* values |
| --- | --- | --- | --- | --- |
| Male | 4493(65.7) | 4457(65.7) | 36(65.5) | 0.965 |
| Age | 64.0(56.0-73.0) | 64.0(56.0-73.0) | 66.0(59.0-75.0) | **0.065** |
| Diastolic pressure on admission | 88(80-97) | 88(80-97) | 87(74-100) | 0.493 |
| Systolic pressure on admission | 150(136-165) | 150(136-165) | 154(130-170) | 0.610 |
| NIHSS score on admission **< 0.001** | | | | |
| 0-5 | 4313(63.1) | 4295(63.3) | 18(32.7) |  |
| 6-15 | 2115(30.9) | 2090(30.8) | 25(45.5) |  |
| >15 | 407(6.0) | 395(5.8) | 12(21.8) |  |
| GCS score on admission **< 0.001** | | | | |
| 13-15 | 6126(89.6) | 6085(89.7) | 41(74.5) |  |
| 9-12 | 538(7.9) | 531(7.8) | 7(12.7) |  |
| 3-8 | 171(2.5) | 164(2.4) | 7(12.7) |  |
| **Past history** | | | | |
| Previous stroke | 1660(24.3) | 1648(24.3) | 12(21.8) | 0.668 |
| Hypertension | 4387(64.2) | 4355(64.2) | 32(58.2) | 0.351 |
| Diabetes mellitus | 1706(25.0) | 1692(25.0) | 14(25.5) | 0.932 |
| Dyslipidemia | 201(2.9) | 200(2.9) | 1(1.8) | 0.621 |
| Coronary heart disease | 994(14.5) | 985(14.5) | 9(16.4) | 0.701 |
| Atrial fibrillation | 426(6.2) | 412(6.1) | 14(25.5) | **< 0.001** |
| History of tumors | 168(2.5) | 164(2.4) | 4(7.3) | **0.021** |
| **Medication before admission (3 months prior to stroke)** | | | | |
| Lipid-lowering agents | 269(3.9) | 266(3.9) | 3(5.5) | 0.561 |
| Antiplatelet agents 0.762 | | | | |
| None | 6257(91.5) | 6206(91.5) | 51(92.7) |  |
| Single antiplatelet agents | 503(7.4) | 500(7.4) | 3(5.5) |  |
| Dual antiplatelet agents | 75(1.1) | 74(1.1) | 1(1.8) |  |
| Antihypertensive agents | 2003(29.3) | 1988(29.3) | 15(27.3) | 0.740 |
| **Treatment in hospital** | | | | |
| Antidiabetic agents | 1971(28.8) | 1953(28.8) | 18(32.7) | **0.523** |
| Antihypertensive agents | 3087(45.2) | 3061(45.1) | 26(47.3) | 0.752 |
| Lipid-lowering agents | 6255(91.5) | 6203(91.5) | 52(94.5) | 0.418 |
| Antithrombotic agents **0.025** | | | | |
| None | 318(4.7) | 313(4.6) | 5(9.1) |  |
| Antiplatelet agents | 5547(81.2) | 5511(81.3) | 36(65.5) |  |
| Anticoagulant agents | 102(1.5) | 100(1.5) | 2(3.6) |  |
| Antiplatelet + anticoagulant agents | 868(12.7) | 856(12.6) | 12(21.8) |  |

Values are reported as n (%) or as Median (interquartile range)

*sICH* symptomatic intracranial hemorrhage, *NIHSS* National Institutes of Health Stroke Scale, *GCS* Glasgow Coma Scale

*p* values in bold fonts indicate significant association

**Table S2** Multivariate analysis to identify factors associated with sICH in patients with ischemic stroke without thrombolysis

|  | OR | 95%CI | *P* values |
| --- | --- | --- | --- |
| Atrial fibrillation | 3.432 | 1.723-6.835 | **< 0.001** |
| History of tumors | 3.255 | 1.128-9.391 | **0.029** |
| NIHSS score on admission | | | |
| 0-5 | *Ref.* |  |  |
| 6-15 | 2.844 | 1.524-5.307 | **0.001** |
| >15 | 5.073 | 1.601-16.072 | **0.006** |

*NIHSS* National Institutes of Health Stroke Scale, *OR* odds ratio, *CI* confidence interval

*p* values in bold fonts indicate significant association

**Table S3** Antithrombotic regimens and sICH in patients with atrial fibrillation

|  | All | Without sICH  （n= 412） | With sICH  (n= 14) | *P* values |
| --- | --- | --- | --- | --- |
| Antithrombotic agents 0.192 | | | | |
| None | 31(7.3) | 28(6.8) | 3(21.4) |  |
| Antiplatelet agents | 201(47.2) | 196(47.6) | 5(35.7) |  |
| Anticoagulant agents | 53(12.4) | 52(12.6) | 1(7.1) |  |
| Antiplatelet + anticoagulant agents | 141(33.1) | 136(33.0) | 5(35.7) |  |

*sICH* symptomatic intracranial hemorrhage

**Table S4** Tumor type and sICH in acute ischemic stroke patients without thrombolysis

|  | All | Without sICH  （n= 164 ） | With sICH  (n= 4) | *P* values |
| --- | --- | --- | --- | --- |
| Nasopharyngeal cancer | 5(3.0) | 5(3.0) | 0(0.0) | 0.251 |
| Malignant tumors of the digestive system | 26(15.5) | 24(14.6) | 2(50.0) |  |
| Lung cancer | 11(6.5) | 11(6.7) | 0(0.0) |  |
| Breast cancer | 12(7.1) | 12(7.3) | 0(0.0) |  |
| Liver cancer | 7(4.2) | 6(3.7) | 1(25.0) |  |
| Reproductive system tumors | 27(16.1) | 27(16.5) | 0(0.0) |  |
| Hematological system tumors | 2(1.2) | 2(1.2) | 0(0.0) |  |
| Other tumors | 73(43.5) | 72(43.9) | 1(25.0) |  |
| Combined with 2 types of tumors | 5(3.0) | 5(3.0) | 0(0.0) |  |

*sICH* symptomatic intracranial hemorrhage

**List of Chinese Acute Ischemic Stroke Treatment Outcome Registry**

**(CASTOR) Investigators:**

**1. Jiangxi Provincial People’s Hospital:** Xiaomu Wu

**2. Tongji Hospital:** Zhiyu Nie

**3. The First Affiliated Hospital of Henan University of Chinese Medicine:** Xiangzhe Liu

**4. Nanshi Hospital of Nanyang City:** Junfeng Shi

**5. The First People’s Hospital of Yunnan Province:** Li Ding

**6. Zhengzhou Central Hospital:** Dai Huang

**7. The First Affiliated Hospital of Fujian Medical University:** Ning Wang

**8. Inner Mongolia Medical University:** Jingfen Zhang

**9. Hiser Hospital of Qingdao:** Ruiyou Guo

**10. Qiqihar First Hospital:** Xuerong Qiu

**11. Peking University Shenzhen Hospital:** Jun Wu

**12. Guangzhou General Military Hospital of the Chinese People's Liberation Army:** Yan Liu

**13. The Fourth Affiliated Hospital of China Medical:** Lianbo Gao

**14. First People's Hospital of Zhengzhou:** Aifan Li

**15. Gongyi People's Hospital:** Yuhui Han

**16. Autonomous Region Hospital of Traditional Chinese Medicine Affiliated to Xinjiang Medical University:** Xinling Meng

**17. Daqing Field General Hospital:** Xuhai Gong

**18. The First Afiliated Hospital of Dalian Medical university:** Jie Han

**19. Beijing Friendship Hospital Affiliated to Capital Medical University:** Yongbo Zhang

**20. Yutian County Hospital of Tangshan City:** Jinzhao Wang

**21. Liqun Hospital of Putuo District:** Shuhong Ju

**22. 202 Hospital of the Chinese People's Liberation Army:** Zhilin Jiang

**23. The Fourth Affiliated Hospital of Harbin Medical University:** Shuyan Zhang

**24. Weihai Municipal Hospital:** Zhenguang Li

**25. Affiliated Central Hospital of Tengzhou:** Deyang Li

**26. The Second People's Hospital of Changzhou City Affiliated to Nanjing Medical:** Wenwei Yun

**27. The Third Affiliated Hospital of Sun Yat-sen University:** Zhengqi Lu

**28. Affiliated Hospital of North Sichuan Medical College:** Juming Yu

**29. The Third Hospital of Hebei Medical University:** Junyan Liu

**30. Tianjin First Central Hospital:** Zhiyun Wang

**31. The Affiliated Hospital of Xuzhou Medical University:** Deqin Geng

**32. The First Hospital of Foshan:** Yukai Wang

**33. Hebei General Hospital:** Peiyuan Lv

**34. Shanghai Ninth People’s Hospital:** Danhong Wu

**35. Renji Hospital:** Yangtai Guan

**36. Qilu Hospital of Shangdong University:** Cuilan Wang

**37. The Second Affiliated Hospital of Guangzhou Medical University:** Qingchun Gao

**38. Second Hospital Affiliated to Xinjiang Medical University:** Xuejun Zhang

**39. Xiyuan Hospital of China Academy of Chinese Medicine Sciences:** Hongmei Liu

**40. Beijing Hospital:** Tao Gong

**41. The 260th Hospital of the Chinese People's Liberation Army:** Shujuan Tian

**42. The Second Affiliated Hospital of Zhejiang University School of Medicine:** Shuijiang Song

**43. Branch of Shanghai General Hospital:** Shaoshi Wang

**44. Capital Medical University Xuanwu Hospital:** Haiqing Song

**45. Wuhan University Renmin Hospital:** Zuneng Lu

**46. The Third People’s Hospital of Hubei:** Xiaoxiang Peng

**47. The First Affiliated Hospital of Soochow University:** Qi Fang

**48. West China Hospital:** Wendan Tao

**49. Jiujiang NO.1 People's Hospital:** Shilie Wang

**50. The First Hospital Affiliated to Xinxiang Medical University:** Ping Zhang

**51. Fushun Third Hospital:** Xiaojie Wang

**52. Fushun Central Hospital:** Qiang Zhang

**53. Harbin NO.4 Hospital:** Weishu Xue

**54. Affiliated Luoyang Central Hospital of Zhengzhou University:** Liping Wei

**55. Huizhou Central People's Hospital:** Weiliang Luo

**56. The First Affiliated Hospital of Jiamusi University:** Yuling Jin

**57. Shengjing Hospital of China Medical University:** Juan Feng

**58. The third affiliated hospital of xinxiang medical university:** Xinyan Wu

**59. The Second Affiliated Hospital of Dalian Medical University:** Yongzhong Lin

**60. The Affiliated Hospital of Jiujiang University:** Hongbing Nie

**61. Donggang Central Hospital:** Wei Liu

**62. Brain Hospital Affiliated to Nanjing Medical University:** HuiCao

**63. Affiliated Donghua Hospital of Sun Yat-sen University:** Qi Tan

**64. The First Affiliated Hospital of Henan University of science and technology:** Yude Zhang

**65. First Hospital of Nanchang:** Youqing Deng

**66. China-Japan Union Hospital of Jilin University:** Guangxian Nan

**67. The First Hospital of Dandong:** Lishu Wan

**68. The Second Affiliated Hospital of Nanchang University:** Xiaoping Yin

**69. Zhuhai People' s Hospital:** Wenyan Zhuo

**70. General Hospital of Jinan Military Command:** Bingzhen Cao

**71. Tangshan worker Hospital:** Yongqiu Li
